# Effect of EGCG-based paste as intracanal dressing, in MMPs 2 and 9 expression in dog’s periapical lesions

**DOI:** 10.1590/0103-6440202405509

**Published:** 2024-03-22

**Authors:** Andiara De Rossi, Tadeu Pradela, Fernanda Souza Liévana, Raquel Assed Bezerra Segato, Jorge Esquiche León, Léa Assed Bezerra da Silva, Paulo Nelson-Filho

**Affiliations:** 1 Department of Pediatric Dentistry, School of Dentistry of Ribeirão Preto, University of São Paulo, Ribeirão Preto, SP, Brazil; 2 Oral Pathology, Department of Stomatology, Public Oral Health, and Forensic Dentistry, School of Dentistry of Ribeirão Preto, University of São Paulo, Ribeirão Preto, SP, Brazil.

**Keywords:** matrix metalloproteinases, EGCG, periapical lesion, immunofluorescence, calcium hydroxide

## Abstract

High expression of MMP-2 and MMP-9 in periapical lesions plays an important role in the degradation of the extracellular matrix. This study aimed to investigate the effect of epigallocatechin-3-gallate (EGCG)-based endodontic paste as an intracanal dressing on the expression of MMP-2 and MMP-9 in periapical lesions. Periapical lesions were experimentally induced in 35 mature beagle dog premolars randomly divided into healthy teeth, untreated periapical lesions, periapical lesions treated in a single session (control groups), and periapical lesions treated in two sessions with EGCG or calcium hydroxide-based pastes (experimental groups). After 120 days, specimens were obtained for histopathologic and immunofluorescence analyses to assess the expression of MMP-2 and MMP-9. The statistical analysis was performed using a p-value of 0.05. Endodontic treatment in two sessions using medication with EGCG and calcium hydroxide-based pastes provided similar repair of the apical and periapical tissues and neoformation of periodontal ligament fibers, cementum, and alveolar bone (p>0.05). The experimental groups treated in two sessions with both medications presented expression of MMP-2 and MMP-9 similar to that in healthy teeth (p>0.05), and significantly lower than teeth treated in a single session or untreated periapical lesions (p <0.001). Expression of MMP-2 and MMP-9 was observed in the cytoplasm of fibroblasts, osteoblasts, cementoblasts, cementocytes, and vascular endothelium. The use of EGCG-based endodontic paste reduced the expression of MMP-2 and MMP-9 and allowed repair of periapical lesions, similar to calcium hydroxide-based paste, and superior to treatment performed in a single session.

## Resumo

A alta expressão de MMP-2 e MMP-9 em lesões periapicais desempenha um papel importante na degradação da matriz extracelular. Este estudo teve como objetivo investigar o efeito de uma pasta à base de epigalocatequina-3-galato (EGCG) como curativo intracanal na expressão de MMP-2 e MMP-9 em lesões periapicais. Lesões periapicais foram induzidas experimentalmente em 35 pré-molares de cães da raça beagle, maduros, divididos aleatoriamente em dentes saudáveis, lesões periapicais não tratadas, lesões periapicais tratadas em uma única sessão e lesões periapicais tratadas em duas sessões com a pasta de EGCG ou pasta à base de hidróxido de cálcio. O operador monitorou os animais e realizou a eutanásia após 120 dias para análises histopatológicas e de imunofluorescência para avaliar a expressão de MMP-2 e MMP-9. A análise estatística foi realizada utilizando p=0,05. O tratamento endodôntico em duas sessões com pasta à base de EGCG e pasta à base de hidróxido de cálcio proporcionou níveis semelhantes de reparação dos tecidos apicais e periapicais e neoformação de fibras do ligamento periodontal, cemento e osso alveolar. Em ambos os grupos, a expressão de MMP-2 e MMP-9 foi mínima, sendo observada no citoplasma de fibroblastos, osteoblastos, cementoblastos, cementócitos e endotélio vascular. Em ambos os grupos tratados em duas sessões, a expressão de MMP-2 e MMP-9 foi semelhante à dos dentes hígidos e significativamente menor do que nas lesões periapicais tratadas em sessão única ou não tratadas (p < 0,001). O uso da pasta à base de EGCG reduziu a expressão de MMP-2 e MMP-9 e permitiu o reparo de lesões periapicais, semelhante à pasta à base de hidróxido de cálcio, e foi superior ao tratamento realizado em sessão única.

## Introduction

Matrix metalloproteinases (MMPs) are a family of more than 25 zinc- and calcium-dependent proteases related to the function and structure of tissues. They are mainly involved in the degradation of protein components of the extracellular matrix (ECM), including dentin and bone ^1^. MMPs are produced by polymorphonuclear leukocytes, keratinocytes, monocytes, macrophages, fibroblasts, and mesenchymal cells, and they may be present in inflammatory cells ^2^. MMPs are most often isolated on the cell surface or inside the extracellular matrix ^3^ and are synthesized as inactive zymogens, which can occur through intracellular, extracellular, or cell surface-mediated proteolytic mechanisms ^4^. MMP expression in healthy tissues is present at low levels; however, in inflammatory and infectious pathological conditions such as periodontal and periapical diseases, MMP levels are elevated ^3^ ^,^ ^4^ ^,^ ^5^.

During the progression of pulpal and periapical diseases, MMP-2 and MMP-9 play an important role in the immune and inflammatory response of the host, promoting the degradation of the extracellular matrix and the maintenance of pulp tissue and periapical inflammation ^4^. High expression of MMP-2 and MMP-9 can be found in periapical lesions and intracanal exudates of teeth with acute periapical abscesses, periapical granulomas and cysts and crevicular gingival fluid of teeth with chronic periapical lesions ^3^ ^,^ ^5^. The increase in MMP expression during periapical lesion development can be reduced by endodontic treatment performed in two visits, with the use of calcium hydroxide intracanal medication following mechanical instrumentation, but not with endodontic treatment performed in a single visit ^5^.

Aiming to reduce toxicity and microbial resistance caused by synthetic drugs, herbal extracts have been proposed in the prevention and treatment of autoimmune, infectious, and inflammatory diseases ^6^. Green tea, derived from *Camellia sinensis*, has been widely studied due to its beneficial effects on general and oral health ^6^ ^,^ ^7^. These effects are due to the presence of epigallocatechin-3-gallate (EGCG), which has proven benefits as an antioxidant, anti-inflammatory, anti-carcinogenic, and antihypertensive agent ^8^ ^,^ ^9^. In addition, green tea has antimicrobial activity ^10^, presenting a high spectrum of action against Gram-positive and Gram-negative bacteria ^11^.

In endodontics, the use of EGCG has been suggested as a medication between sessions ^12^ ^)^ or as an irrigant of root canals ^13^. Due to its strong antioxidant properties, the use of EGCG as a final irrigant may neutralize any residual sodium hypochlorite and oxygen and increase the bond strength of root canal sealers ^14^ ^)^ and bonding systems to root dentin ^15^. Recently, studies carried out by our research group developed a formulation based on EGCG for endodontic use that presented tissue compatibility and promoted the healing of periapical tissues ^12^, and the teeth treatment post radiotherapy with EGCG inactivates the enzymatic activity of MMPs activated by radiotherapy ^16^. Thus, it appears that in addition to playing an anti-inflammatory, antioxidant, antimicrobial, and tissue regenerator role, intracanal medication with EGCG may inactivate MMPs. However, the effects of EGCG-based paste on the reduction of MMP-2 and MMP-9 in AP were not suggested or demonstrated *in vivo* after topical use. Thus, this study aimed to investigate the effect of intracanal dressing epigallocatechin-3-gallate (EGCG)-based paste on the expression of MMP-2 and MMP-9 during the repair of periapical lesions. This investigation hypothesizes that EGCG dressing in association with mechanical root canal treatment would reduce the expression of MMPs 2 and 9, reduce inflammation and tissue resorption, and enhance the repair of periapical tissues.

## Materials and methods

### Formulation composition

An EGCG derived from green tea and available on 5 μm provided in the solid-state (E41430; Sigma-Aldrich; St Louis, MO, USA) was used. The EGCG-based paste for endodontic use was prepared as previously reported ^12^ at a 1 mg/mL concentration using polyethylene glycol 400 (PEG 400, Galena Química e Farmacêutica Ltda., Campinas, SP, Brazil) as a vehicle and zinc oxide (2 g) (SS White Artigos Dentários Ltda., Rio de Janeiro, RJ, Brazil) as a radiopacity agent. A calcium hydroxide paste (Calen®, S.S. White Dental Articles Ltda.) was used for comparison because it is currently the most common medication used during endodontic treatment. This paste is comprised of 2.5 g calcium hydroxide and 0.05 g colophony, with 0.5 g zinc oxide as a radiopacity agent and 2 mL polyethylene glycol 400 (PEG 400) as a vehicle.

### Animals

All animal procedures were performed while conforming to protocols reviewed and approved by the Animal Care Committee of the University of São Paulo (Protocol #11.1.1405.53.8). The studies also followed standards recommended by the International Organization for Standardization (ISO) n^o^ 7405/2018 ^17^, except the recommended experimental periods (28 and 90 days), aiming to restrict the number of animals to the minimum necessary to obtain conclusive results, in accordance with Brazilian law and ARRIVE guideline 2.0 for reporting animal research ^18^.

Four beagle dogs of both genders at 12 months of age and 33 pounds were selected. For the experiment, the 2nd and 3rd upper premolars and the 2nd, 3rd, and 4th lower premolars were used. In cases of gingivitis and/or gingival calculus, the animals received prophylaxis, scraping, straightening, and dental polishing, followed by the application of 0.12% chlorhexidine digluconate (Periogard - Colgate - Palmolive - Indústria Ltda. - Brazil).

### Operative procedures

The same operator performed all procedures. Animals were pre-anesthetized and anesthetized intravenously with zolazepam. For induction of periapical lesions after crown access, pulp tissue was removed and root canals were left exposed to the oral cavity for 7 days to allow microbial contamination ^19^. Afterward, cavities were sealed with zinc oxide-eugenol cement (IRM^®^, Dentsply Industria e Comércio LTDA - Petrópolis - Brazil). Standardized periapical radiographs were taken until the development of periapical radiolucencies, which occurred after 45 days.

After this period, the teeth were isolated with a rubber dam, and disinfection of the operative field was made with 2% chlorhexidine gluconate. The working length was determined at 1 mm short of the radiographic apex and confirmed by periapical radiography. The apical delta was perforated by using #20 to #25 K-files at the length of the tooth to create a standardized apical opening. The root canals were instrumented by the ProTaper Universal rotary system (DentsplyMaillefer, Ballaigues, Switzerland) in the sequence recommended by the manufacturer (S1 0.18/.02, S2 0.20/.04, F1 0.20/.07, F2 0.25/.08, F3 0.30/.09, F4 0.40/.06 and F5 0.50/.05) using the XSmart^TM^ endodontic micromotor (Dentsply Maillefer Instruments; Ballaigues, Switzerland) under irrigation with 3.6 mL 2.5% NaOCl at each file change. The ethylene-diamine-tetra-acetic acid (EDTA) solution was used as a penultimate wash for 3 minutes under agitation with a k file followed by a final rinse with NaOCl solution.

Five groups were formed according to the control or experimental conditions (Table 1). The sample size was calculated by G*Power software using previous studies ^5^ ^,^ ^12^ ^,^ ^19^ ^,^ ^20^ ^,^ ^26^ ^,^ ^27^, a p-value of 0.05, and a power calculation of 0.08.

**Table 1:** Groups evaluated and the distribution of the number of roots

Clinical control and experimental conditions and treatments	Number of roots
Healthy tooth (control)	10 roots
Tooth with untreated periapical lesion (control)	10 roots
Tooth treated endodontically in a single session (control)	10 roots
Tooth treated endodontically in two sessions: paste based on EGCG (experimental)	20 roots
Tooth treated endodontically in two sessions: paste based on calcium hydroxide (experimental)	20 roots

1. Healthy control group (healthy and nontreated teeth): MMP-2 and MMP-9 expression in healthy tissues was characterized.
2. Untreated control group (teeth with untreated periapical lesions): MMP-2 and MMP-9 expression in periapical lesions experimentally induced and nontreated endodontically was characterized.
3. One session control group (teeth with induced periapical lesions submitted to endodontic treatment performed in a single session): the root canal filling was finished in the same session after chemomechanical preparation.
4. EGCG experimental group (teeth with induced periapical lesions submitted to endodontic treatment performed in two sessions with EGCG-based intracanal dressing): root canal filling was performed 14 days after the application of endodontic medication.
5. Calcium hydroxide experimental group (teeth with induced periapical lesions submitted to endodontic treatment performed in two sessions with calcium hydroxide-based intracanal dressing): The root canal filling was applied 14 days after the application of endodontic medication.

In the EGCG and calcium hydroxide experimental groups, the paste was applied at 1 mm beyond the working length to promote a very slow extrusion of the medication into the periradicular tissues, which was radiographically assessed. This procedure was performed with the aid of an ML threaded syringe (S.S. White Artigos Dentários Ltda.; Rio de Janeiro, Brazil) and a long needle 27G (Septoject XL; Septodont, France). Sealing was achieved with glass-ionomer-based cement for 14 days. At the end of this period, the operator removed the intracanal dressing by irrigation and performed root filling using the AH Plus sealer (De Trey; Dentsply, Konstanz, Germany) and gutta-percha cones by lateral condensation with a final radiographic confirmation. All teeth were restored with a base of glass-ionomer cement and silver amalgam.

### Histotechnique processing

After 120 days of the first section of endodontic treatment animals were euthanized. The maxilla and mandible were removed, dissected, sectioned, fixed, washed, and subjected to decalcification. Subsequently, the pieces were neutralized, washed, dehydrated in alcohol, cleared in xylol, and embedded in paraffin. Serial longitudinal sections 5μm-thick were cut in mesiodistal orientation. For histopathological analysis, the sections were initially stained with hematoxylin and eosin (H&E), and evaluated by conventional light microscopy (Leica DMR, Leica Microsystem Wetzlar Gmbh; Wetzlar, Germany).

Microscopic analysis was performed by two examiners, with the Kappa (K) concordance test at k = 0.81 without prior knowledge of the group to which the analyzed specimen belongs. They evaluated the integrity of the extracellular matrix and the presence and intensity of the inflammatory infiltrate on slides stained with H&E under conventional and fluorescent light microscopy. An assessment of the degree of tissue disorganization was carried out to characterize the repair stage through the reinsertion and neoformation of the fibers of the periodontal ligament attached to the apical third. The presence or absence of periodontal ligament fibers attached to cementum and the degree of collagen fiber disruption and disorganization at apical and periapical region was classified as absent, (score 1), mild (score 2), moderate (score 3), or severe (score 4) to indicate the level of periodontal ligament destruction as previously reported ^5^. The attribution of each score was based on a previous broad survey of all specimens without prior knowledge of the group belonging ^5^. This criterion was also used to assess the integrity or level of resorption of the adjacent cementum and alveolar bone.

### Immunofluorescence processing

To evaluate the expression of matrix metalloproteinases and the distribution of these enzymes in tissues (pulpal, apical, and periapical region), immunofluorescence assays were performed for MMP-2 and MMP-9.

The slides were deparaffinized, hydrated in a decreasing series of alcohols, and washed under running water. The antigenic recovery with Proteinase K 1: 500 (Invitrogen, Carlsbad, USA) was performed for 10 minutes. The slides were washed in phosphate-buffered saline (PBS) for 5 minutes (3 times). After that, sodium borohydride 1mg / mL (Dinâmica Química Contemporânea LTDA, Indaiatuba, Brazil) was applied for 15 minutes (3 ×), the slides washed again in PBS for 5 minutes (3x) and the nonspecific binding sites blocked with 1% bovine serum albumin (Sigma, St Louis, USA) for 60 minutes. The tissues were incubated with the primary antibodies in 1:50 concentration for MMP-2 (5 g / mL; 53630, Santa Cruz Biotechnology, Dallas, USA) and MMP-9 (5 g / mL; 21736, Santa Cruz Biotechnology) at 4 ºC overnight. Then, the slides were removed from the refrigerator and placed at room temperature for 1 hour. After that, they were washed in PBS for 5 minutes (3 times), incubated with biotinylated secondary anti-mouse FITC antibodies at a concentration of 1: 200 for 1 hour (Rabbit anti-mouse IgG-FITC sc-358916, Santa Cruz Biotechnology), washed in PBS for 5 minutes (3 times), and finally, the coverslips were placed using an Aqueous Mounting Medium with DAPI (UltraCruz^®^24941, Santa Cruz Biotechnology, Dallas, USA. Control slides were used to test the specificity of the immunostaining in which the primary antibody was omitted and the slides were incubated in phosphate-buffered saline (PBS).

The microscopic analysis was performed by two examiners with the Kappa (K) concordance test at k = 0.83, without prior knowledge of the group to which the analyzed specimen belongs.

### Statistical analysis

The quantitative histopathological results (tissue disorganization, inflammatory infiltrate, and MMP-2 and MMP-9 expression) are ordinal, independent variables with three different categories and sizes for the experimental and control groups. The normal distribution of data was assessed using the Shapiro-Wilk test. Due to the non-normal distribution of the data, for each variable, the Kruskall-Wallis nonparametric test was used, followed by Dunn's post-test for multiple comparisons. The statistical software used was SPSS version 25 (IBM, Chicago, USA) with a significance level of 5% (p < 0.05).

## Results

### Descriptive Histological Analysis

**Figure 1:**
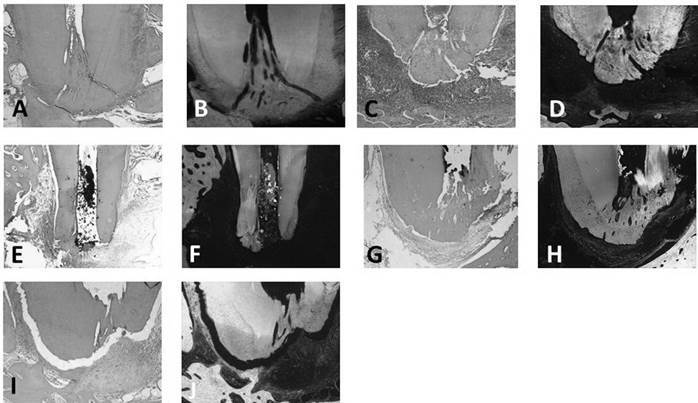
Representative microscopic images of dogs apical and periapical tissues stained with HE and evaluated under conventional light microscopy (left images) and fluorescence microscopy (right images). A and B: healthy tooth, C and D: tooth with untreated periapical lesions; E and F: periapical lesions endodontically treated in a single session; G and H: periapical lesions endodontically treated in two sessions with EGCG-based paste and I and J: periapical lesions endodontically treated in two sessions with calcium hydroxide-based paste. Magnification 5X.

**Figure 2:**
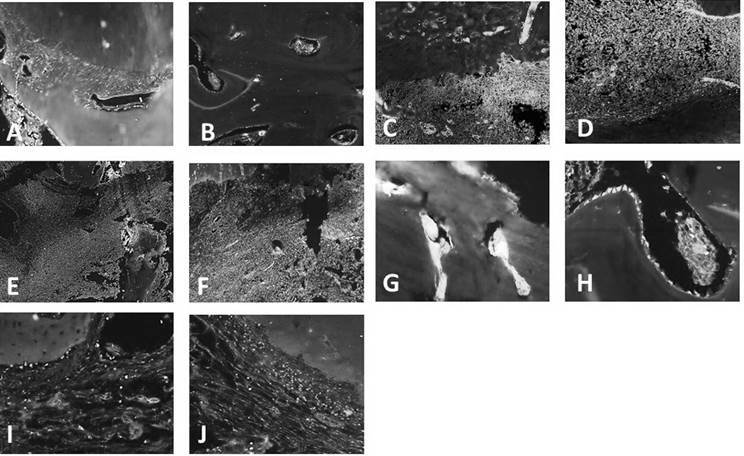
Representative microscopic images from immunofluorescence staining of MMP-2 (left images) and MMP-9 (right images) in dogs' apical and periapical tissues. A and B: healthy tooth showing positive and mild expression of MMP-2 in membrane and cytoplasm of fibroblasts at the periodontal ligament space, cementoblasts, and osteoblasts, and MMP-9 in osteoblasts present in mature bone. C and D: tooth with untreated periapical lesions presenting positive and severe immunostaining of MMP-2 and MMP-9 in cementocyte lacunae, inflammatory infiltrate cells, and cementoblasts. E and F: periapical lesions endodontically treated in a single session presented severe expression of MMP-2 and MMP-9 in persistent cells of inflammatory infiltrate and cementocyte lacunae. G and H: periapical lesions treated with EGCG-based paste presented mild expression of MMP-2 and MMP-9 located in cementocytes, cementoblasts, and osteoblasts lining repaired alveolar bone surface. I and J: periapical lesions treated with calcium hydroxide-based paste showed a positive and mild expression of MMP-2 and MMP-9 in cementoblasts and fibroblasts. Fluorescence microscopy. Magnification: A, C-F: 10X; B 20X; G-J: 40X.

In the Healthy Group, the intact premolars presented pulp vitality in the coronary and root regions, integrity of the apical and periapical tissues, and absence of inflammatory infiltrate (Figure 1. A-B). Positive and mild expression of MMP-2 and MMP-9 (score 2) was observed, located in the fibroblast membrane and cytoplasm between the fibers of the periodontal ligament (Figure 2. A), cementoblasts and osteoblasts lining cement and bone surface. MMP-9 expression in osteoblasts was present in mature bone, with evidence of the transition process from newly formed bone and lamellar bone during the physiological process of bone remodeling (Figure 2. B).

The teeth of the Untreated Group, where induction of periapical lesions was not followed by endodontic treatment, presented pulp necrosis in the entire length of the root canal associated with the presence of a chronic inflammatory infiltrate, composed of polymorphonuclear and mononuclear cells, in the apical and periapical region, associated to resorption of the fibers of the periodontal ligament, resorption of external and internal surface of cementum with absence of cementocytes in some lacunaes. The resorption of the alveolar bone surrounding the periapical lesion was also observed (Figure 1. C-D). There was a positive and severe immunostaining of MMP-2 and MMP-9 in the periapical lesion, located inside the cementocyte’s lacunae and in the cells of the inflammatory infiltrate and cementoblasts (Figures 2. C-D).

After endodontic treatment performed in a one-session Group, without the application of endodontic dressing, the persistence of a severe inflammatory infiltrate and resorption of mineralized tissues, including cementum and alveolar bone and absence of repair of periodontal ligament fibers was observed (Figure 1. E-F). There was a positive and severe expression of MMP-2 and MMP-9 in the periapical lesion, similar to teeth with untreated periapical lesions (p>0.05). Expression of MMP-2 and MMP-9 can be seen in the cells of the inflammatory infiltrate throughout the periapical lesion and inside the cementocyte lacunae still resorbed as a consequence of the inflammatory response (Figure 2. E-F).

In the EGCG Group, the teeth treated in two sessions by the use of EGCG dressing before root canal filling, it was possible to visualize the repair of periapical lesions, with the reestablishment of the periodontal ligament space with reinsertion of collagen fibers, bone, and cement neoformation, and absence of inflammatory infiltrate (Figure 1. G-H). In this group, a mild expression of MMP-2 and MMP-9 was observed in the periapical region, similar to healthy tissues (Figure 2. G-H). A significant reduction of the immunofluorescent expression of MMP-2 and MMP-9 was observed after endodontic treatment using an EGCG-based paste compared to the expression of MMP-2 and MMP-9 in the teeth with untreated periapical lesions or treated in one-session (p<0.05).

In the Calcium Hydroxide Group, it was also possible to observe the repair process of periapical lesions, with reinsertion of collagen fibers at periodontal ligament, neoformation of alveolar bone, and cementum at external and internal root surface and absence of inflammatory infiltrate (Figure 1. I-J). A positive and mild expression of MMP-2 and MMP-9 was observed, similar to the group treated with EGCG-based paste (p>0.05), with a significant reduction of its immunofluorescent expression when compared to teeth of Untreated and One-session Groups (p<0.001). It was also possible to visualize the immunostaining of MMP-2 and MMP-9 in cells such as odontoblasts and cementoblasts, which configure the participation of these proteases in physiological processes of bone tissue repair and tissue repair of the root cementum (Figure 2. I and 2. J).

### Quantitative analysis by scores

The results of the quantitative histological analyses (tissue disorganization, inflammatory infiltrate, and expression of MMP-2 and MMP-9) are listed in Table 2.

**Table 2:** Percentage of roots (n) for each score, according to a histological analysis performed by different clinical conditions and after different protocols of endodontic treatment.

Quantitative histological parameters	Scores	Clinical conditions and treatments				
		Healthy Tooth	Untreated Periapical Lesion	Single Session	EGCG-based paste	Calcium hydroxide-based paste
Tissue disorganization	Absent	100	0	0	80	85
	Mild	0	0	0	20	15
	Moderate	0	0	30	0	0
	Severe	0	100	70	0	0
	*Statistical analysis*	*b*	*a*	*a*	*b*	*b*
Inflammatory Infiltrate	Absent	100	0	0	80	90
	Mild	0	0	0	20	10
	Moderate	0	0	30	0	0
	Severe	0	100	70	0	0
	*Statistical analysis*	*b*	*a*	*a*	*b*	*b*
Expression of MMP-2	Absent	0	0	0	0	0
	Mild	100	0	0	85	80
	Moderate	0	0	40	15	20
	Severe	0	100	60	0	0
	*Statistical analysis*	*b*	*a*	*a*	*b*	*b*
Expression of MMP-9	Absent	0	0	0	0	0
	Mild	90	0	0	80	85
	Moderate	10	0	30	20	15
	Severe	0	100	70	0	0
	*Statistical analysis*	*b*	*a*	*a*	*b*	*b*

There were no statistically significant differences between untreated or single-visit treated teeth (p>0.05), which presented severe scores in most of the specimens for all parameters evaluated: tissue disorganization, inflammatory infiltrate, and expression of MMP-2 and MMP-9. There were also no differences between periapical lesions treated with EGCG and calcium hydroxide (p>0.05) that presented most of the specimens with the absence of tissue disorganization and inflammatory cells infiltration and mild expression of MMP-2 and MMP-9, similar to healthy teeth (p>0.05). Teeth with untreated periapical lesions or with periapical lesions treated in a single session were different from teeth treated in two sessions with EGCG or calcium hydroxide and healthy teeth (p<0.01).

## Discussion

The results of the present study confirm the hypothesis that the intracanal dressing with EGCG-based pastes following root canal instrumentation reduced the expression of MMPs 2 and 9 and favored the repair of apical and periapical tissues, allowing neoformation of periodontal ligament fibers, cementum and alveolar bone and reducing inflammation. During endodontic treatment, the healing of apical periodontitis after the use of calcium hydroxide-based paste has been already proved in several studies ^5^ ^,^ ^20^ but this study is the second to show repair after the use of EGCG ^12^. In addition to previous studies, the present immunohistochemical study shows that the use of EGCG and calcium hydroxide similarly promotes the reduction of the expression of MMP-2 and MMP-9 in periapical lesions, in levels very similar to that of periapical tissues of healthy teeth.

The expression of MMPs has been found in association with the Toll-like receptors 2 and 4 in symptomatic and asymptomatic apical periodontitis, which may explain the clinical presentations and the evolution of apical periodontitis and may represent key targets for new diagnostic and treatment approaches ^21^. Furthermore, the present study allowed the identification of positive staining for MMPs 2 and 9 in cells such as odontoblasts, osteoblasts, cementoblasts, cementocytes, fibroblasts, and blood vessels, which shows the important participation of these enzymes in tissue repair processes of apical periodontitis. It was reported that EGCG inhibited the formation and differentiation of osteoclasts via inhibition of MMPs 2 and 9 in rats ^22^. In addition, EGCG could prevent the alveolar bone resorption that occurs in periodontal diseases, by inhibiting the expression of MMP-9 in osteoblasts and the formation of osteoclasts ^23^. Other studies also show the immunostaining of MMP-2 and MMP-9 in osteoblasts, however, in the respective studies, rats and mice were used as an experimental animal model ^22^ ^,^ ^23^. In the present beagle dogs were used as models for the histopathological study of apical periodontitis due to its anatomical and physiopathological similarities with humans. Experimental induction of apical periodontitis in dog’s teeth has been performed by histopathological studies for evaluation of new endodontic materials and modalities of treatment under the same operatory conditions applied in humans and with healing responses obtained in shorter periods than humans ^5^ ^,^ ^12^ ^,^ ^19^ ^,^ ^20^ ^,^ ^25^ ^,^ ^26^ ^,^ ^27^. The difference was the present study was carried out after 120 days and not 180 days aiming to reduce the period of animal experimentation since the radiographic follow-up has already shown conclusive results of periapical lesion repair at 120 days, as also observed in previous studies of our research group ^12^ ^,^ ^26^.

The clinical significance of the present study is that periapical lesion repair following endodontic treatment is impacted by therapy performed either in a single-visit by mechanical instrumentation or multiple visits by mechanical instrumentation followed by intracanal medication with EGCG or calcium hydroxide-based pastes. The present study showed that teeth endodontically treated in a single session presented severe expression of MMP-2 and MMP-9 2 in the periapical region, persistence of inflammatory infiltration and tissue disorganization and resorption, similar to periapical lesions experimentally induced and not submitted to endodontic treatment. The expression of MMP-9 and transforming growth factor beta (TGF-β1) was previously evaluated in samples of periapical lesions and correlated with the intensity of the inflammatory infiltrate and the thickness of the epithelial lining, showing that the extracellular matrix remodeling process is dependent on MMP-9 appears to be similar for periapical granulomas and root cysts ^24^. In addition, MMP-2 and MMP-9 play a critical role in the development of periapical inflammatory lesions, probably involved in the degradation of the extracellular matrix (ECM) during the early stage of development of periapical injury in rats ^4^.

The differences in the histopathological repair of periapical lesions observed between teeth endodontically treated in a single session or two sessions may be attributed to differences in the reduction of intra- and extraradicular infection. It is well known that the use of an endodontic medication that presents tissue compatibility, including its intentionally slow extrusion into the periradicular tissues, may improve eradication of root canal system infection, especially extraradicular infection and periapical biofilms, that may not be affected by chemomechanical procedures, and such persistence is associated with failures of endodontic treatment and the maintenance of lesions ^2^.

A limitation of the present study is related to the two sessions of endodontic treatment, which allowed for the use of more irrigation during the second visit, which may also favor the resolution of the infection present inside the root canal. The irrigation method may interfere with the reduction of intraradicular endodontic infection. In the present study conventional irrigation was performed but previous *in vivo* histopathological studies have suggested better revascularization and periapical repair after endodontic treatment using apical negative pressure irrigation versus conventional irrigation intracanal dressing in dogs' immature teeth ^25^ and mature teeth with apical periodontitis ^26^. Thus, considering that intra- and extraradicular biofilm removal and inactivation may be accomplished by both chemomechanical processes and intracanal medicaments, further clinical, radiographic, microbiologic, and histopathological studies should be performed to assess the repair of periapical lesions using different irrigation activation systems performed in one or two sessions and associated or not with intracanal medications with EGCG or calcium hydroxide.

The use of EGCG-based paste as an intracanal dressing resulted in a reduction in the expression of MMPs 2 and 9 and repair of periapical lesions very similar to those obtained by the use of a calcium hydroxide-based paste. A point to be considered is that the pure form of EGCG, extracted from the *Camellia sinensis* plant used in the present study, is commercialized in powder form and needs to be prepared by adding vehicle and radiopacity before use. Therefore, a limitation of using EGCG-based paste for endodontic use is its high costs in comparison to calcium hydroxide and its non-existence on the market since there is still no commercially available EGCG endodontic formulation. On the other hand, in addition to presenting tissue compatibility, based on its antimicrobial action against several species involved in endodontic infection and inhibition of the proteolytic activity of MMPs, the antioxidant role of EGCG presents double effects on neutralizing the side effects of sodium hypochlorite and increasing bond stability and adhesion filling sealers ^20^ ^,^ ^21^. Thus, additional studies are required to evaluate the extensive properties of EGCG, including its potential to act as a biological modifier of the internal root canal surface and its involvement in other signaling pathways or tissue inhibitors of MMPs 2 and 9, which can be evaluated with different methodologies that can be applied during endodontic treatment and repair of mineralized and nonmineralized tissues.

It can be concluded that the EGCG-based paste used as an intracanal dressing in dogs resulted in a reduction of the immunofluorescent expression of MMP-2 and MMP-9, and the histopathologic repair of periapical lesions, similar to the calcium hydroxide-based paste and superior to the treatment of teeth with periapical lesion performed in a single session.
